# Overgrazing on unmanaged grassland interfered with the restoration of adjacent grazing-banned grassland by affecting soil properties and microbial community

**DOI:** 10.3389/fmicb.2023.1327056

**Published:** 2024-01-04

**Authors:** Mengchao Fang, Guang Lu, Shuping Zhang, Wei Liang

**Affiliations:** ^1^College of Life and Environment Science, Minzu University of China, Beijing, China; ^2^Ministry of Education Key Laboratory for Ecology of Tropical Islands, Key Laboratory of Tropical Animal and Plant Ecology of Hainan Province, College of Life Sciences, Hainan Normal University, Haikou, China

**Keywords:** grazing ban, overgrazing, restoration, soil properties, soil microbial community, edge effect

## Abstract

A “grazing ban” policy has been implemented in some pastoral areas in China to fence degraded grasslands for restoration. However, fencing increased grazing pressures in unmanaged grasslands. Based on the mechanism of negative edge effect, we investigated whether overgrazing on unmanaged grassland interfered with the restoration of adjacent grazing-banned grassland by affecting soil properties and microbial community using a sample in Hulun Buir of Inner Mongolia, in order to optimize the “grazing ban” policy. Plant and soil were sampled in areas 30 m away from the fence in unmanaged grassland (UM) and in areas 30 m (adjacent to UM) and 30–60 m (not adjacent to UM) away from the fence in the grazing-banned grassland (F-30 m and F-60 m). The species richness and diversity of plant communities and the ASV number of fungal communities significantly decreased in F-30 m and UM, and the Simpson index of the bacterial community significantly decreased in F-30 m compared with F-60 m. The abundance of fungi involved in soil organic matter decomposition significantly decreased and the abundance of stress-resistant bacteria significantly increased, while the abundance of bacteria involved in litter decomposition significantly decreased in UM and F-30 m compared with F-60 m. The simplification of plant communities decreased in soil water and total organic carbon contents can explain the variations of soil microbial communities in both UM and F-30 m compared with F-60 m. The results of PLS-PM show that changes in plant community and soil microbial function guilds in UM may affect those in F-30 m by changing soil water and total organic carbon contents. These results indicate that overgrazing on unmanaged grassland interfered with the restoration of adjacent grazing-banned grassland by affecting soil properties and microbial community. The grazing-banned grasslands should be adjusted periodically in order to avoid negative edge effects.

## 1 Introduction

Grassland is an ecosystem where humans and nature coexist and interact (Wang et al., 2018; Sun et al., 2022). Grasslands provide grazing resources for humans, and moderate grazing promotes decomposition and regulates the structure of plant communities, maintaining the dynamic balance of grasslands (Xun et al., 2018). However, the ongoing impact of overgrazing and global climate change has resulted in grassland degradation, which has become a major factor threatening the stability of the global terrestrial ecosystem (Boval and Dixon, 2012; Gibbs and Salmon, 2015; Liu et al., 2019; Lyu et al., 2020; Bardgett et al., 2021; Török et al., 2021). To curb grassland degradation, a series of restoration policies have been implemented in some pastoral areas of China (Kolås, 2014; Sun et al., 2020). “Grazing ban” is one such policy to fence some grasslands from grazing. The implementation of the policy significantly improved the plant diversity, total organic carbon, soil water content, and total nitrogen in the grazing-banned areas (Pei et al., 2008; Cheng et al., 2016; Wang et al., 2016; Xiong et al., 2016; Li et al., 2017). However, fencing increased grazing pressure in the unmanaged areas which are usually adjacent to the grazing-banned areas (Sun et al., 2020). In some long-term fenced grasslands, the areas adjacent to unmanaged grasslands have degraded. Therefore, it is necessary to understand whether the overgrazing on unmanaged grasslands interfered with the restoration of grazing-banned grasslands.

Fencing formed a clear boundary between unmanaged and grazing-banned grasslands. Edge effect refers to the changes in community structure and ecological processes caused by ecological factors or system-attributed changes at the edge zone of two adjacent ecosystems (Fonseca and Joner, 2007; Eldegard et al., 2015). In anthropogenic interference-induced edge zones, the biological community structures within the edge zones changed rapidly and had weak anti-interference abilities (Kark, 2013; Smith and Goetz, 2021). Light, energy utilization rate, soil temperature, and soil physicochemical properties in such areas changed with the changes in the biological community (Schmidt et al., 2017; Koelemeijer et al., 2023). Thus, edge effects induced by anthropogenic interference have been confirmed to negatively influence species diversity, community dynamics, and ecosystem functions (Guerra et al., 2017; Krishnadas et al., 2018; Fischer et al., 2021; Blanchard et al., 2023; Lapola et al., 2023).

It is well known that the anthropogenic interference-induced edge effect is caused by disrupting the interaction between soil microbial and plant communities in the edge zone (Pennanen, 2001; Ettema and Wardle, 2002; Malmivaara-Lämsä et al., 2008). The interaction between plants and soil microorganisms is important for maintaining the stability and productivity of ecosystems (van der Heijden et al., 2008; Bardgett and van der Putten, 2014). Plants serve as specific hosts for soil microorganisms (Zhang et al., 2018), influence the microbial community structure through root exudates (Meier et al., 2017; Canarini et al., 2019), and provide energy and nutrients for microbial metabolism (van der Heijden et al., 2008; Luo et al., 2022). Soil microorganisms regulate nutrient cycling between plants and soil, aiding the formation of soil structures that are conducive to plant growth (Bissett and Burke, 2007; Coban et al., 2022; Hartmann and Six, 2023; Li et al., 2023). The soil microbial community structure and function in the edge zone change with the changes in soil properties and plant community, and these changes influence litter decomposition rates and nutrient cycling in the edge zone (Pennanen, 2001). Changes in nutrient and water supply may in turn affect the structure and diversity of plant communities in adjacent areas, ultimately leading to vegetation degradation in these areas (Ettema and Wardle, 2002). For example, forest fragmentation-induced edge effect changed soil microbial communities by affecting the soil properties in the forest edge zone (Malmivaara-Lämsä et al., 2008). Based on the mechanism of edge effect induced by anthropogenic interference, overgrazing on unmanaged grassland may deteriorate the interaction between soil microbial and plant communities in adjacent grazing-banned grassland, reducing the restoration effectiveness in grazing-banned grassland. Therefore, it is necessary to understand whether such a negative edge effect exists in order to optimize the “grazing ban” policy.

The Inner Mongolia grassland is one of the pastoral areas in China where the “grazing ban” policy is implemented (Hao et al., 2014; Kolås, 2014; Gao et al., 2022). Grasslands designated by the local government as under “restoration” cover approximately 70% of the total grassland. Of the total area under “restoration”, pastures fenced under “grazing ban” cover approximately 20%, and pastures subjected to unspecified restoration measures cover approximately 20% (Kolås, 2014). Due to the reduction in grazing grasslands, herders overgrazed on the unmanaged grasslands, resulting in severe degradation of these areas. Many such unmanaged areas are adjacent to grazing-banned areas. Based on the known mechanisms of edge effect induced by anthropogenic interference described above, this study aims to investigate whether overgrazing on unmanaged grasslands interfered with the restoration of adjacent grazing-banned grassland by affecting soil properties and microbial communities. We predicted that (1) in grazing-banned grassland, the composition and the interaction of soil microbial and plant communities of the area adjacent to unmanaged grassland were similar to those of the unmanaged grassland but different from those of the area not adjacent to the unmanaged grassland; and (2) the changes of plant and soil microbial communities in the unmanaged grassland may change those in adjacent grazing-banned grassland by changing soil properties.

## 2 Materials and methods

### 2.1 Study site

The study site was located in the Xin Barag Right Banner of Hulun Buir, in the Inner Mongolia Autonomous Region (47°36′00″N ~ 49°50′0″N and 115°31′00″E ~ 117°43′00″E), and is an example of a typical temperate grassland. The average annual precipitation is 243.9 mm, with the majority of rainfall occurring during the summer months. The average annual temperature is 1.6°C. The primary soil type is Calcic Luvisols (FAO, 1988), and the dominant plant species include *Leymus chinensis, Stipa capillata*, *Poa annua,* and *Caragana stenophylla*, among others.

### 2.2 Experimental design

**Figure 1:**
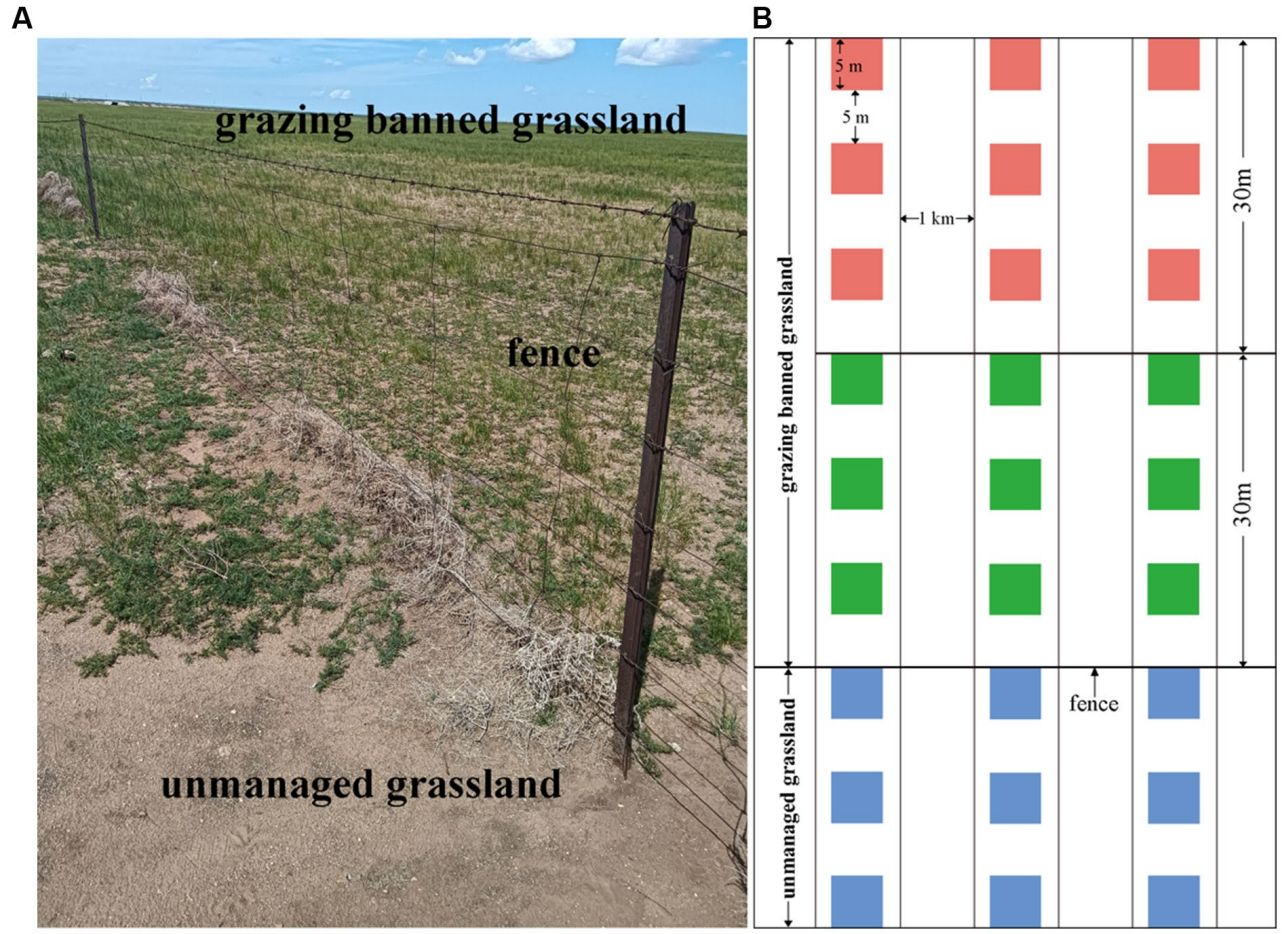
The sample site **(A)** and experimental design diagram **(B)**.

The grazing-banned grassland selected in this study was fenced in 2008 and is adjacent to an unmanaged grassland (Figure 1A). Three belt transects spaced 1 km apart were set up perpendicular to the fence. According to the observation that degradation area in the grazing-banned grassland is approximately 30 m away from the fence, we defined 30 m as a distance unit from the fence (Figure 1A). In each belt transect, three 5 m × 5 m plots, spaced 5 m apart, were set up 30 m away from the fence in unmanaged grassland (hereafter UM) and the areas 30 m (adjacent to the unmanaged grassland) and 30–60 m (not adjacent to the unmanaged grassland) away from the fence in grazing-banned grassland (hereafter F-30 m and F-60 m), respectively. The sampling diagram is shown in Figure 1B. Within each plot, the plant community was surveyed, and the soil was sampled. To test our two predictions, we compared the composition of the plant and soil microbial communities, soil microbial functions, and soil physicochemical properties in the three types of sampling grasslands, and then we analyzed the relationships between plant community, soil physicochemical properties, and soil microbial community in UM and F-30 m, respectively; finally, we estimated the direct and indirect effects of plant community, soil property, and soil microbial community in UM on those in F-30 m.

### 2.3 Plant community survey and soil sampling

The plant community survey and soil sampling were conducted in July 2022. Plant species were identified, and the number of each species in each plot was counted. Five soil samples, 5 cm in diameter and 20 cm deep, were taken from the four corners and centers of each plot. Samples collected from the same plot were combined and passed through a 2 mm sieve before being divided into three parts for analysis. One portion was stored at 4°C for analysis of pH and soil water content (SWC). Another portion was air dried for analysis of soil element content, and the final portion was stored at −80°C for Illumina sequencing of soil microorganisms.

### 2.4 Analysis of soil physicochemical properties

The soil samples were immediately weighed after collection, before being placed in an oven for 24 h at 60°C and weighed again. The difference between the wet and dry soil weights was used to determine the soil water content (SWC). The soil pH values were measured in the soil suspension (soil:water = 1:2.5) using a pH meter (Sartorius PB-10, Gottingen, Germany). We measured the total organic carbon (TOC) and total nitrogen (TN) contents using a carbon-nitrogen analyzer (Vario Max CN, Elementar, Germany), total phosphorus (TP) content using ammonium molybdate spectrophotometry, and available potassium (AK) content using flame atomic absorption spectroscopy.

### 2.5 Soil microbial analysis

We extracted DNA from different samples using Omega Bio-Tek (Soil DNA Kit D5625, United States), amplified the V4 region of the bacterial 16S rRNA gene using 515F (5’-GTGYCAGCMGCGGTAA-3′) and 806R (5’-GACTACHVGGGTWTCTAAT-3′) primers, and then amplified the ITS2 region of fungi using ITS1FI2 (5’-GTGARTCATC GAATCTTTG-3′) and ITS2 (5’-TCCTCTTATTGC-3′) primers. PCR amplification was performed in a 25 μl reaction mixture containing 50 ng template DNA, 12.5 μL Phusion Hot start flex 2X Master Mix, and 2.5 μL forward and reverse primers, and ddH_2_O was used to adjust the volume to 25 μL. The PCR amplification conditions were as follows: initial denaturation at 98°C for 30 s, followed by 32 cycles consisting of denaturation at 98°C for 10 s, annealing at 54°C for 30 s, and extension at 72°C for 45 s. The bacteria and fungi were amplified 35 times and 32 times, respectively, followed by a final extension step at 72°C for 10 min. PCR products were purified and quantified using AMPure XT beads (Beckman Coulter Genomics, Danvers, MA, United States) and Qubit (Invitrogen, United States), respectively. Amplicon library was prepared, and its size and quantity were assessed using an Agilent 2,100 Bioanalyzer (Agilent, United States) and the Library Quantification Kit for Illumina (Kappa Biosciences, Woburn, MA, United States). Finally, we used the NovaSeq PE250 platform to sequence the library.

### 2.6 Bioinformatics analysis on microbial community

We separated the sample data based on barcode information, removed joints and barcode sequences, and then concatenated and filtered the data. The Differential Amplicon Denoising Algorithm 2 (DADA2) was used for length filtering and denoising, and single base precision biological sequences were obtained and renamed as amplified subsequence variants (ASVs), and singleton ASVs were removed. The feature sequences of each ASV were obtained through taxonomic annotation. Based on the obtained ASV feature sequence and ASV abundance table, the Shannon and Simpson indices of bacteria and fungi in each sample were calculated, and the dominant phyla were determined (relative abundance >1%). The prediction of the functional guilds of bacteria and fungi was performed in FAPROTAX and FUNGuild, respectively.

### 2.7 Calculation

We defined the number of plant species in each sample plot as plant species richness and measured plant species diversity using the Shannon−Weiner index (Spellerberg and Fedor, 2003). Based on the Z-score of the average abundance of each plant species, a cluster analysis was carried out on the composition of plant species. The microbial community alpha diversity was indicated by the Shannon−Weiner and Simpson indices (Wang et al., 2020). Based on the Bray−Curtis distance, we performed clustering analyses on the composition of microorganisms at the phylum and genus levels. We calculated the abundance of dominant phyla and functional guilds of bacteria and fungi.

### 2.8 Statistical analysis

One-way ANOVA and Tukey’s HSD multiple comparison tests were used to determine the significance of differences in the soil physicochemical properties and the alpha diversity index of plant communities among different types of grassland. The Kruskal–Wallis test and Steel–Dwass multiple comparison tests were used to determine the significance of differences in the alpha diversity index of microbial community and abundance of dominant phyla and functional guilds of soil microbial community among different types of grassland. Redundant analysis (RDA) was used to identify the effects of plant community composition and soil physicochemical properties on the soil microbial community composition in UM and F-30 m, respectively, taking F-60 m as a reference group. Pearson correlation was used to analyze the relationship between plant community composition, soil physicochemical properties, and the relative abundance of functional guilds of microorganisms in UM and F-30 m, respectively. Finally, we used partial least squares path modeling (PLS-PM) to estimate the influence paths among plant communities, soil properties, and soil microbial communities in UM and F-30 m. The PLS-PM (“plspm” package in R) used PLS regression to estimate the direction and strength of linear correlations between multiple variables. GoF > 0.5 is used as the indicator to determine the fitness of a path model. One-way ANOVA and Kruskal–Wallis tests were completed in SPSS 20.0, while RDA analysis, Pearson correlation, and PLS-PM were conducted in R4.2.2.

## 3 Results

### 3.1 Plant and soil microbial community composition and soil physicochemical properties

**Figure 2:**
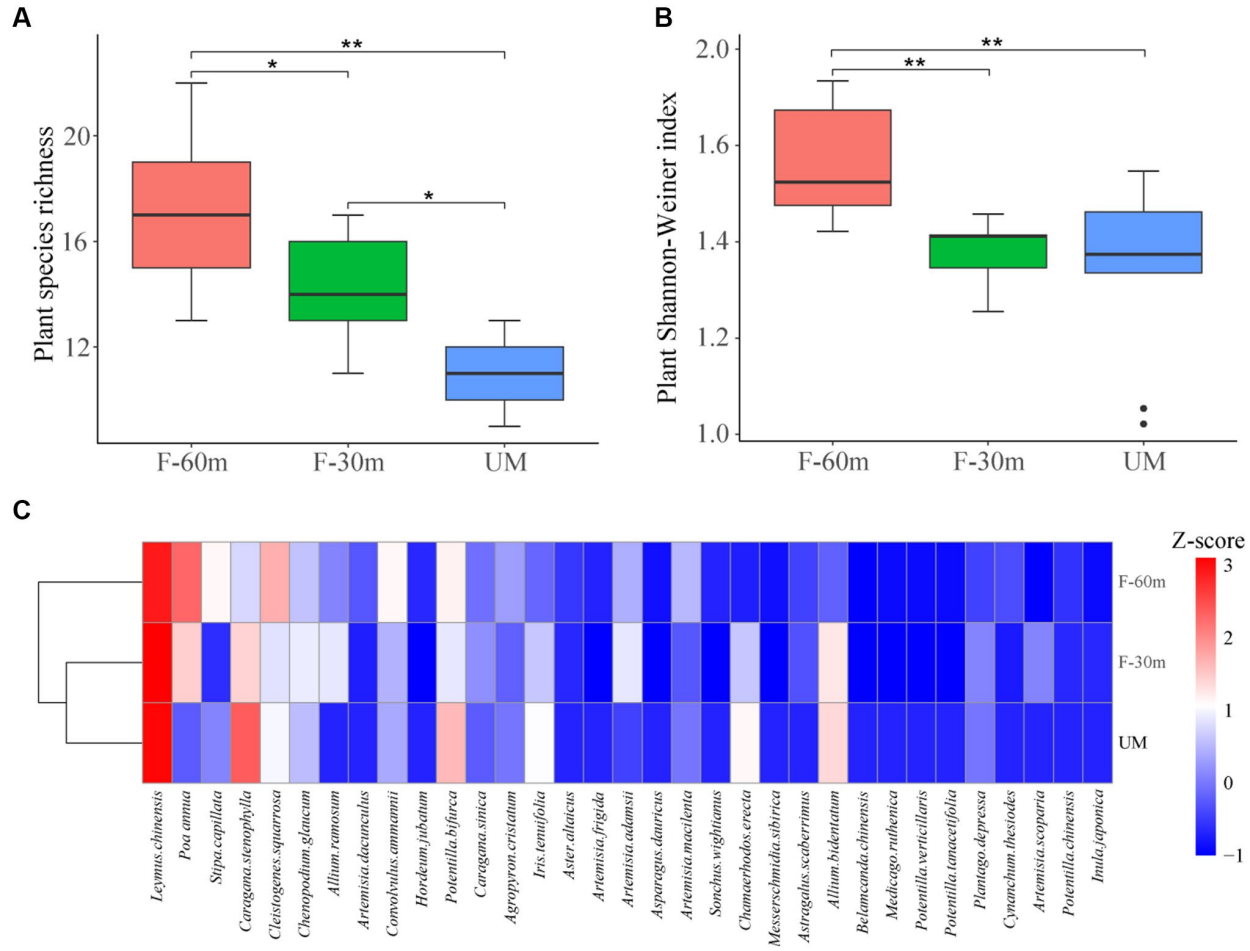
Plant species richness **(A)**, Shannon–Weiner Index **(B)**, and cluster analysis **(C)** of unmanaged grassland (UM) and the areas 30 m and 30–60 m away from the fence in grazing-banned grassland (F-30 m and F-60 m). ** and * mean one-way ANOVA and Tukey’s HSD test, *p* < 0.01 and *p* < 0.05. Z-score is the average abundance of each plant species.

**Figure 3:**
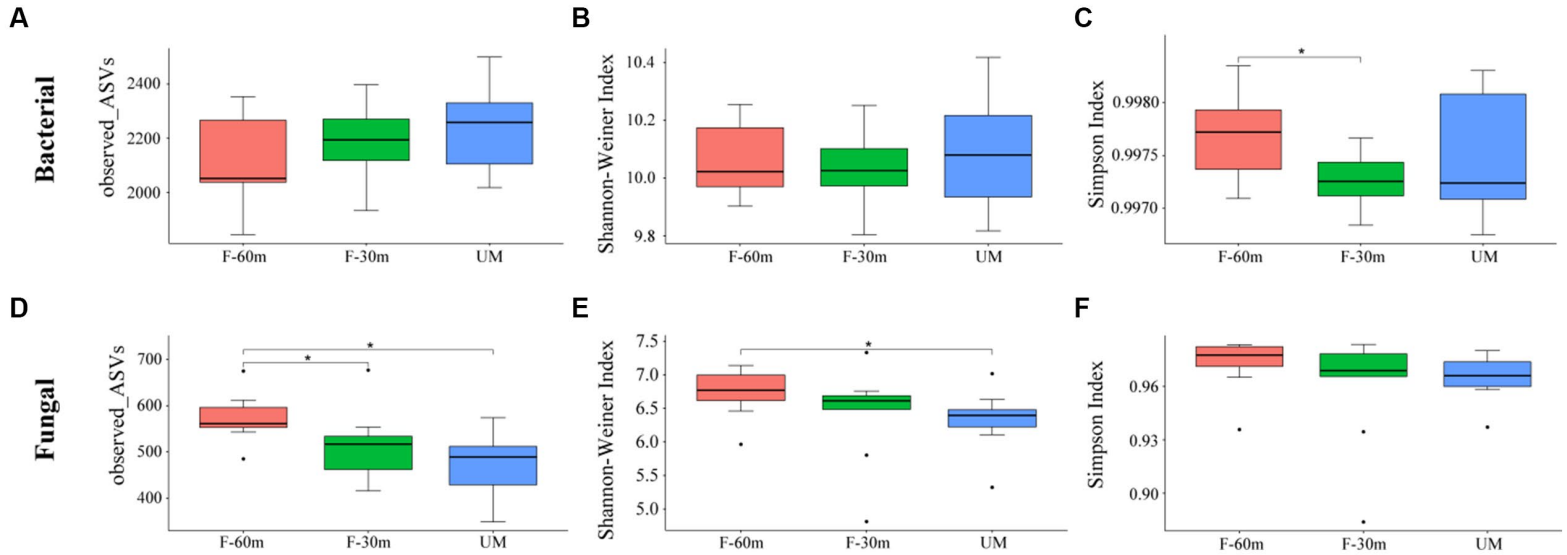
The alpha diversity of bacteria and fungi in unmanaged grassland (UM) and the areas 30 m and 30–60 m away from the fence in grazing-banned grassland (F-30 m and F-60 m). * means Kruskal–Wallis and Steel–Dwass tests *p* < 0.05. **(A)** bacterial ASV number; **(B)** bacterial Shannon–Weiner index; **(C)** bacterial Simpson index; **(D)** fungal ASV number; **(E)** fungal Shannon–Weiner index; **(F)** fungal Simpson index.

**Figure 4:**
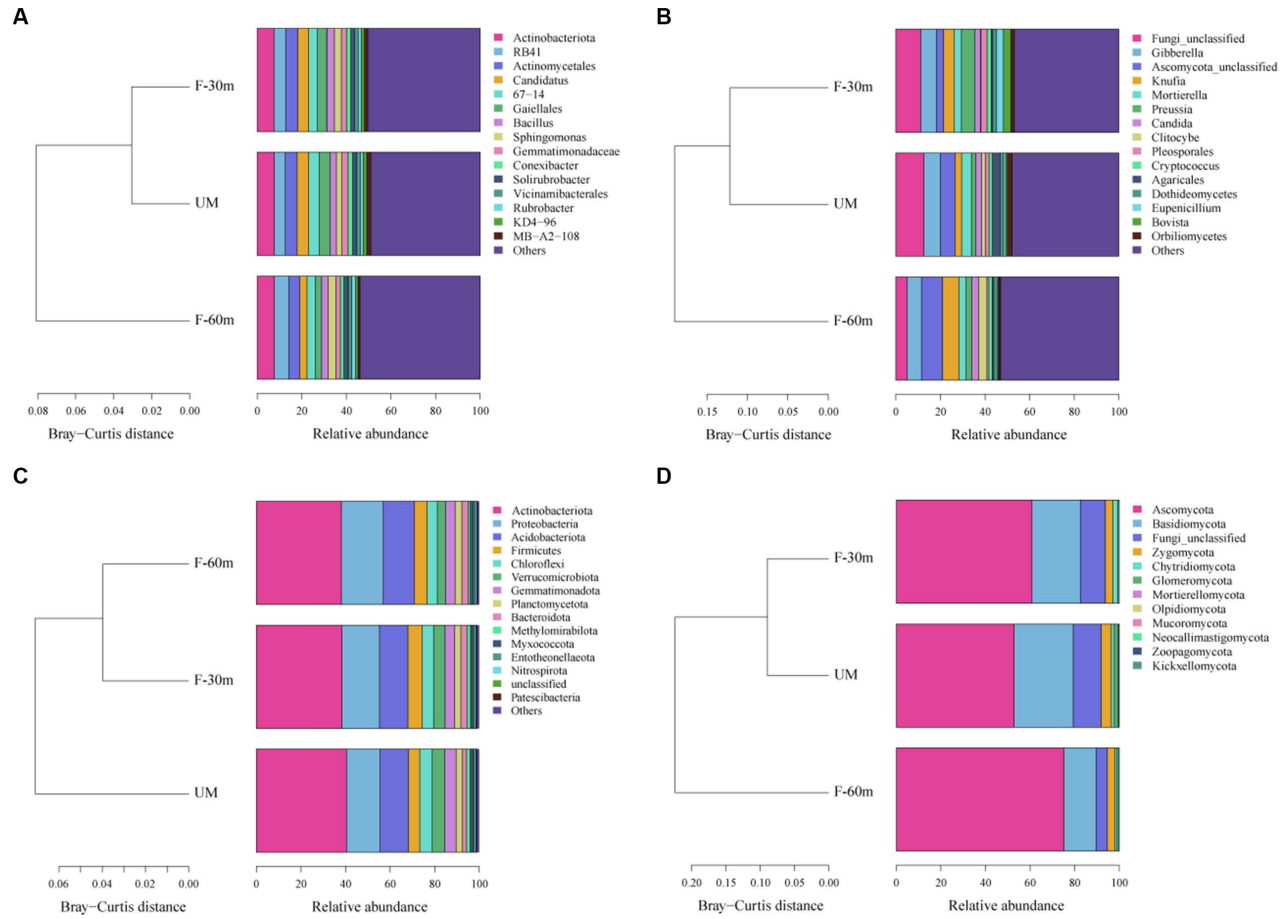
Cluster analyses based on Bray–Curtis distance for dominant bacterial genera **(A)**, fungal genera **(B)**, bacterial phyla **(C)**, and fungal phyla **(D)** in unmanaged grassland (UM) and the areas 30 m and 30–60 m away from the fence in grazing-banned grassland (F-30 m and F-60 m).

**Figure 5:**
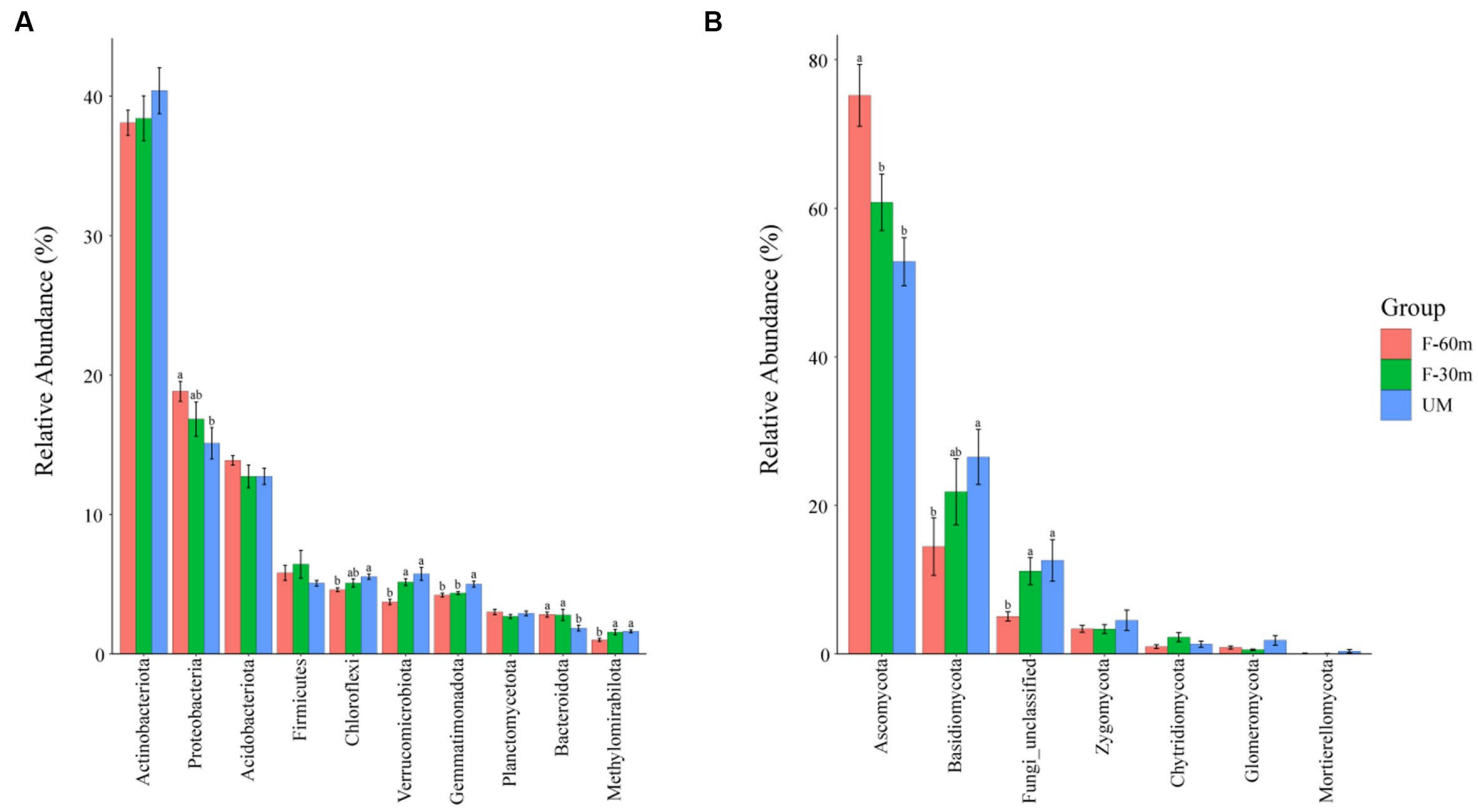
The relative abundance of bacterial dominant phyla **(A)** and fungal dominant phyla **(B)** in unmanaged grassland (UM) and the areas 30 m and 30–60 m away from the fence in grazing-banned grassland (F-30 m and F-60 m). The relative abundance of dominant phyla is mean ± standard deviation. The lowercase letters (a, b) on the bar chart indicate significant differences between different grasslands (Kruskal–Wallis and Steel–Dwass tests, *p* < 0.05).

Plant species richness and Shannon–Weiner index were significantly lower in F-30 m and UM than in F-60 m (one-way ANOVA and Tukey’s HSD test, *p* < 0.05; Figures 2A,B). *Leymus chinensis*, *Caragana stenophylla, Poa annua*, and *Cleistogenes squarrosa* are the dominant species in three types of grassland (Figure 2C). According to the results of cluster analysis, there were more Gramineae plants in F-60 m than in F-30 m and UM (Figure 2C). The Simpson index of bacterial community in F-30 m significantly decreased (Kruskal–Wallis and Steel–Dwass tests, *p* < 0.05, Figure 3C). The numbers of ASVs of fungal communities in UM and F-30 m significantly decreased (Kruskal–Wallis and Steel–Dwass tests, *p* < 0.05, Figure 3D). The Shannon–Weiner index of fungal community significantly decreased in UM (Kruskal–Wallis and Steel–Dwass tests, *p* < 0.05, Figure 3E). The bacteria genus, fungal genus, and phylum in F-30 m are similar to UM and different from F-60 m (Figures 4A,C,D), while the bacteria phylum in F-30 m is similar to F-60 m and different from UM (Figure 4B). There was no significant difference in the numbers of ASVs and Shannon–Weiner index of bacterial community and Simpson index of fungal community among different types of grassland (Figures 3A,B,F, Kruskal–Wallis, *p* > 0.05). The abundances of Verrucomirobiota and Methlomirabiota significantly increased, but the abundance of Proteobacteria significantly decreased in UM and F-30 m (Kruskal–Wallis and Steel–Dwass tests, *p* < 0.05, Figure 5A). The abundances of Chloroflexi and Bacteroidota significantly increased and decreased in UM, respectively (Kruskal–Wallis and Steel–Dwass tests, *p* < 0.05, Figure 5A). The abundances of Ascomycetes and Fungi_unclassified in UM and F-30 m significantly decreased and increased, respectively (Kruskal–Wallis and Steel–Dwass test, *p* < 0.05, Figure 5B). The abundance of Basidiomycota significantly increased in UM (Kruskal–Wallis and Steel–Dwass tests, *p* < 0.05, Figure 5B). The TOC content and SWC in UM and F-30 m significantly decreased. The TN content significantly decreased in F-30 m. The TP content significantly increased in UM (one-way ANOVA and Tukey’s HSD test, *p* < 0.05, Table 1).

**Table 1:** Soil physicochemical properties in the unmanaged grassland (UM) and the areas 30 m and 30–60 m away from the fence in grazing-banned grassland (F-30 m and F-60 m).

	F-60 m	F-30 m	UM
SWC (%)	8.76 ± 041^a^	7.53 ± 0.44^b^	7.64 ± 0.48^b^
TOC (g/kg)	15.08 ± 1.83^a^	11.18 ± 2.90^b^	12.34 ± 2.36^b^
TN (g/kg)	2.03 ± 0.19^a^	1.66 ± 0.23^b^	1.84 ± 0.33^ab^
TP (g/kg)	0.39 ± 0.03^b^	0.38 ± 0.04^b^	0.44 ± 0.05^a^
pH	6.97 ± 0.42^a^	7.12 ± 0.74^a^	6.82 ± 0.55^a^
AK (g/kg)	0.93 ± 0.12^a^	0.85 ± 0.14^a^	0.94 ± 0.060^a^

Soil water content (SWC), total organic carbon (TOC), total nitrogen (TN), total phosphorus (TP), pH, and available potassium (AK) were expressed as mean ± standard deviation. Letters (a, b) indicate significant differences between different grasslands (one-way ANOVA and Tukey’s HSD test, *p* < 0.05).

### 3.2 Soil microbial functional guilds

**Figure 6:**
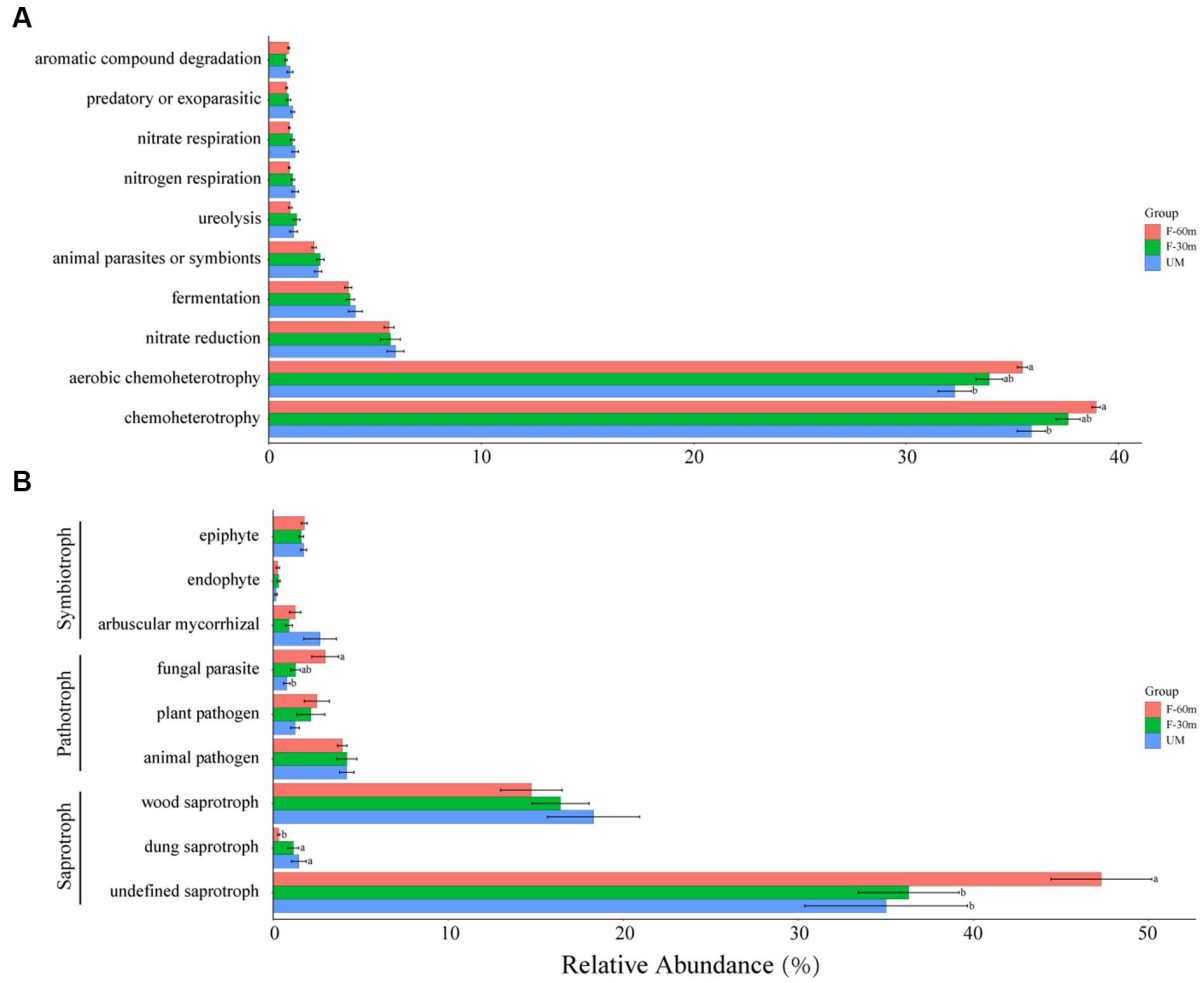
The relative abundance of bacterial functional guilds **(A)** and fungal functional guilds **(B)** in unmanaged grassland (UM) and the areas 30 m and 30–60 m away from the fence in grazing-banned grassland (F-30 m and F-60 m). The relative abundance of functional guilds is mean ± standard deviation. The lowercase letters (a, b) on the bar chart indicate significant differences between different grasslands (Kruskal–Wallis and Steel–Dwass tests, *p* < 0.05).

Among the bacterial functional guilds, the abundance of chemoheterotrophy and aerobic chemoheterotrophy in UM was significantly lower than in F-60 m (Kruskal–Wallis and Steel–Dwass tests, *p* < 0.05; Figure 6A). Among the fungal functional guilds, the abundance of undefined saprotroph in F-30 m and UM was significantly lower than in F-60 m (Kruskal–Wallis and Steel–Dwass tests, *p* < 0.05), while the abundance of dung saprotroph was significantly higher in F-30 m and UM than in F-60 m (Kruskal–Wallis and Steel–Dwass tests, *p* < 0.05). The abundance of fungal parasites in F-60 m was significantly higher than in UM (Kruskal–Wallis and Steel–Dwass tests, *p* < 0.05, Figure 6B).

### 3.3 Effects of plant community and soil properties on soil microbial community

**Figure 7:**
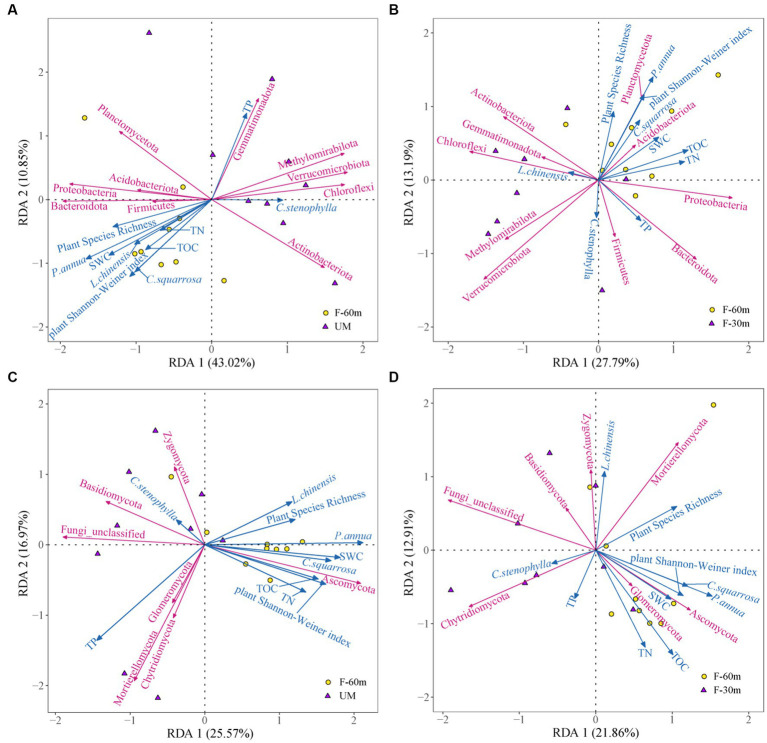
Redundancy analysis on the effect of plant diversity, plant dominant species abundance, and soil properties on soil bacterial communities in UM **(A)** and F-30 m **(B)** and soil fungal communities in UM **(C)** and F-30 m **(D)** compared with F-60 m.

Plant species richness, plant Shannon–Weiner index, SWC, and the abundance of *L. chinensis*, *P. annua,* and *C. squarrosa* were the main factors affecting the bacterial community composition in UM compared with F-60 m, which explained 53.87% of the variation in the composition of the bacterial communities (Figure 7A). Plant Shannon–Weiner index, SWC, TN, and the abundance of *P. annua* were the main factors affecting the bacterial community composition in F-30 m compared with F-60 m, which explained 40.98% of the variation in the composition of the bacterial communities (Figure 7B). Plant Shannon–Weiner index, SWC, TOC, TN, and the abundance of *P. annua* and *C. squarrosa* were the main factors affecting the fungal community composition in UM compared with F-60 m, which explained 42.54% of the variation in the composition of the fungal communities (Figure 7C). Plant Shannon–Weiner index, TOC, TN, and the abundance of *P. annua* and *C. squarrosa* were the main factors affecting the fungal community composition in F-30 m compared with F-60 m, which explained 34.77% of the variation in the composition of the fungal communities (Figure 7D).

### 3.4 Effects of plant community and soil properties on soil microbial functional guilds

**Figure 8:**
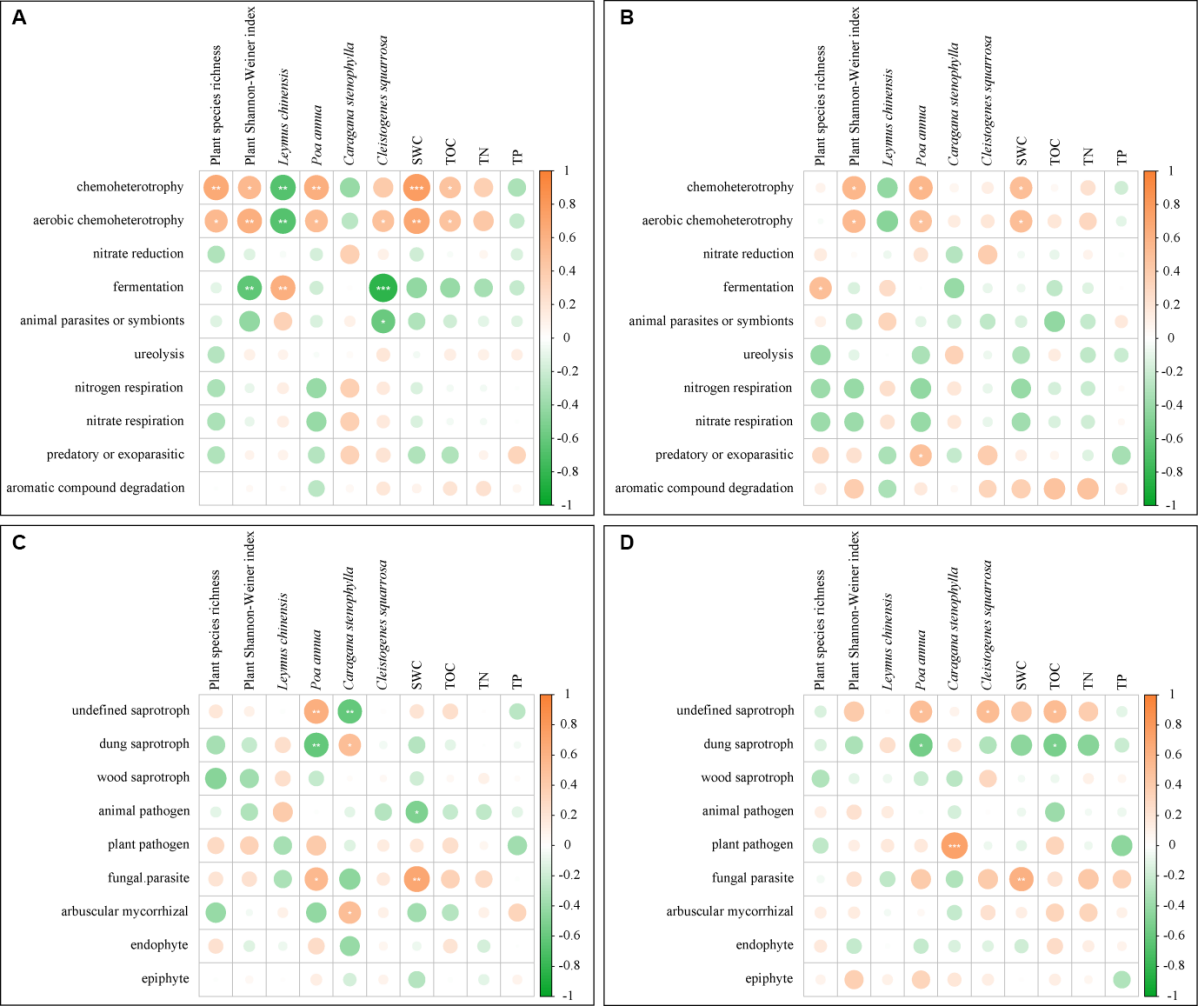
The Pearson correlation between the bacterial and fungal functional guilds and plant diversity, relative abundance of dominant plants, and soil physicochemical properties [**(A)** bacterial guilds in UM and F-60 m samples; **(B)** bacterial guilds in F-30 m and F-60 m samples; **(C)** fungal guilds in UM and F-60 m samples; **(D)** fungal guilds in F-30 m and F-60 m samples]. SWC, soil water content; TOC, total organic carbon; TN, total nitrogen; and TP, total phosphorus. * *p* < 0.05, ** *p* < 0.01, and *** *p* < 0.001.

In UM and F-60 m, plant species richness, plant Shannon–Weiner index, SWC, and TOC positively correlated with the abundance of chemoheterotroph and aerobic chemoheterotroph bacteria (*p* < 0.05, Figure 8A). Three plant species positively or negatively correlated with the abundance of different bacteria functional guilds (*p* < 0.05, Figure 8A). SWC positively correlated with the abundance of parasite fungi (*p* < 0.05, Figure 8C). Two plant species positively or negatively correlated with the abundance of different fungal functional guilds (*p* < 0.05, Figure 8C). In F-30 m and F-60 m, plant Shannon–Weiner index and SWC positively correlated with the abundance of chemoheterotroph and aerobic chemoheterotroph bacteria (*p* < 0.05, Figure 8B). TOC positively and negatively correlated with the abundance of undefined saprotroph fungi and dung saprotroph fungi, respectively (*p* < 0.05, Figure 8D); SWC positively correlated with the abundance of parasite fungi (*p* < 0.05, Figure 8D). Three plant species positively or negatively correlated with the abundance of different fungal functional guilds (*p* < 0.05, Figure 8D).

### 3.5 Path model of the plant community, soil microbial community, and soil properties in UM and F-30 m

**Figure 9:**
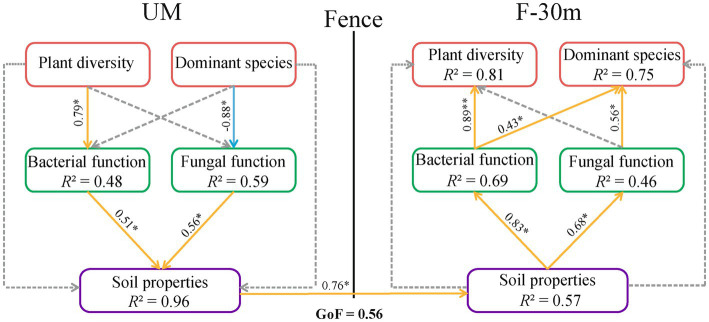
The partial least squares path model (PLS-PM) of the plant diversity, the abundance of dominant species, soil microbial function, and soil properties in UM and F-30 m. The orange and blue arrows indicate positive and negative effects (*p* < 0.05), respectively. The dashed line represents a non-significant (*p* > 0.05) relationship. Plant diversity includes plant species richness and Shannon–Weiner index; dominant species include *Leymus chinensis*, *Caragana stenophyll*, *Poa annua,* and *Cleistogenes squarrosa*; Soil properties include SWC (soil water content) and TOC (total organic carbon).* means *p* < 0.05.

According to the results in 3.4, we selected factors that significantly affect soil microbial function guilds to construct three variables, including plant diversity (plant species richness and Shannon–Weiner index), dominant species abundance (*Leymus chinensis*, *Caragana stenophyll*, *Poa annua,* and *Cleistogenes squarrosa*), and soil properties (SWC and TOC). The PLS-PM estimation was performed between these three variables and soil microbial functions. The path model with GoF = 0.56 is shown in Figure 9. Plant diversity in UM had a positive effect on the bacterial function in UM, while the abundance of dominant species in UM had a negative effect on the fungal function in UM. The bacterial and fungal functions in UM had positive effects on soil properties in UM. The soil properties in UM had a direct positive effect on the soil properties of F-30 m. The soil properties in F-30 m had positive effects on bacterial and fungal functions in F-30 m. Bacterial function in F-30 m had positive effects on plant diversity and dominant species abundance in F-30 m. Fungal function in F-30 m had a positive effect on the abundance of dominant species in F-30 m.

## 4 Discussion

Numerous studies have demonstrated that long-term repeated grazing can reduce plant diversity and vegetation productivity (Eldridge and Delgado-Baquerizo, 2017; Xun et al., 2018). The results showed that UM had significantly lower plant richness and diversity, indicating the degradation of unmanaged areas. The results of the plant Shannon index and species cluster analysis showed that the plant community in F-30 m was similar to that in UM and different from that in F-60 m (Figure 2), indicating that the area adjacent to UM in the grazing-banned grassland exhibited similar degradation to unmanaged grassland, although there was no grazing in this area. Plant community degradation can cause significant changes in microbial communities (Zhou et al., 2019; Wang et al., 2022). The results of fungi ASVs, Shannon index, and cluster analysis showed that the fungi community in F-30 m was also similar to UM and different from F-60 m. The bacterial community at the genus level in the F-30 m was similar to UM. The abundance of stress-resistant microorganisms significantly increased in UM and F-30 m areas, such as Verrucomirobiota and Methylomirabilota, appearing in drought and hypoxic environments (Chowdhury et al., 2019; Zhu et al., 2022). The abundance of bacteria phylum Proteobacteria involved in litter decomposition and fungal phylum Ascomycota involved in degrading lignin and cellulose (Fierer et al., 2007; Wang et al., 2020; Zheng et al., 2021) significantly decreased in UM and F-30 m areas. These results support our first prediction that the structures of plant and soil microbial communities in the areas adjacent to unmanaged grassland in grazing-banned grassland are similar to those in unmanaged grassland but different from those in the areas not adjacent to unmanaged grassland.

The bacterial functional guild analysis reveals that the levels of chemoheterotroph and aerobic chemoheterotroph, which are involved in the transformation of organic matter into soil (Lian et al., 2021; Zheng et al., 2023), are significantly lower in UM and F-30 m than in F-60 m. Thus, the decomposition of soil organic matter in the unmanaged grassland and the edge area in the grazing-banned grassland is weak, which is detrimental to the soil carbon cycling in these areas. The fungal parasite and saprophytic fungal guilds contributed to the majority of differences observed among the three types of grassland. The significant decrease in fungal parasites in UM and F-30 m is related to a decrease in plant diversity, which leads to a decrease in the host population (Maurice et al., 2021). There was a significant decrease in the abundance of undefined saprotrophs in UM and F-30 m. Saprophytic fungi play a crucial role in organic matter decomposition, facilitating the cycling of key soil nutrient elements, affecting species coexistence by altering soil nutrients, and maintaining stable vegetation productivity (Crowther et al., 2012; Chen et al., 2019; Liu et al., 2022). The bacterial and fungal functional guild analysis jointly suggest that soil nutrient cycling in F-30 m and UM may be inhibited.

Long-term overgrazing can reduce plant diversity, dominant species abundance, soil nutrients, and water content in grassland (Wu et al., 2014; Ji et al., 2022; Luo et al., 2022), which is reflected by the plant Shannon–Weiner index, SWC, TOC, and TN reduction in the UM grassland in our study. Human interference can negatively alter soil properties in edge areas, such as TOC, TN, and C/N (Malmivaara-Lämsä et al., 2008). Our results showed that the SWC, TOC, and TN in the F-30 m are similar to those in UM, which indicate that the soil properties in the grazing-banned area adjacent to unmanaged grassland degrade. Degradation in both plant community and soil properties can alter the soil bacterial and fungal composition (Parton et al., 1995; Manzoni et al., 2012; Steinauer et al., 2015; Zhou et al., 2019; Huang et al., 2021; Wang et al., 2021; Yu et al., 2021), especially reducing the microorganisms related to nutrient cycling (Pennanen, 2001; Malmivaara-Lämsä et al., 2008). Changes in plant diversity can alter the composition and function of soil microorganisms by affecting litter input (Steinauer et al., 2015). SWC can directly affect the transformation of soil nutrients and soil microbial metabolism, thereby affecting the composition and function of soil microbial communities (Parton et al., 1995; Manzoni et al., 2012). TOC can provide energy and carbon sources for soil microorganisms (Zhou et al., 2019; Wang et al., 2021). In our RDA and Pearson correlation results, compared with F-60 m, the plant Shannon–Weiner index and the abundance of *Poa annua* influence soil microbial composition and functional guilds in both UM and F-30 m, while the soil TOC and SWC influence the soil microbial functional guilds in both UM and F-30 m. The decrease in plant Shannon–Weiner index, *Poa annua*, TOC, and SWC in UM and F-30 m results in the decrease in chemoheterotroph and aerobic chemoheterotroph bacteria, saprotroph, and parasite fungi in UM and F-30 m, which may reduce nutrient cycling efficiency. These results indicate that the grazing-banned area adjacent to unmanaged grassland exhibits a degraded interaction between plant and microbial communities just like unmanaged grassland.

It has been known that plant community degradation can reduce the abundance of microorganisms related to nutrient cycling and degrade the soil properties of the adjacent area (Pennanen, 2001; Malmivaara-Lämsä et al., 2008). Changes in soil water and nutrient supply may in turn affect the structure and diversity of plant community and ultimately lead to the vegetation degradation of the adjacent area (Ettema and Wardle, 2002). Our PLS path model indicates that plant diversity or dominant species in UM can affect the soil water and organic carbon contents in UM by affecting the bacterial and fungal functional guilds, and the changes of soil properties in UM influence the soil properties in F-30 m. Subsequently, the changes in soil properties in F-30 m affect the bacterial and fungal functional guilds in F-30 m and then affect the plant diversity and dominant species in F-30 m. These results support our second prediction that the degradation of plant community, soil properties, and soil microbial community in unmanaged grassland interfere with soil microbial communities and, consequently, the plant restoration in the adjacent grazing-banned grassland. Such edge effects may gradually interfere with more areas in grazing-banned grassland.

In summary, our results showed that soil microbial composition, soil properties, and plant community composition in the grazing-banned grassland adjacent to unmanaged grassland are similar to those in degraded unmanaged grassland, indicating that the degradation of unmanaged grasslands can affect the restoration of the grazing-banned grasslands via negative edge effect. In our study area, the grazing-banned grassland has been fenced for 15 years. Long-term fencing increased grazing pressure in unmanaged grasslands, and degradation in these areas interfered with the restoration of fenced areas. Traditional nomadism in the Inner Mongolia grassland is distinguished by mobile grazing which helps prevent grassland degradation caused by long-term sedentary grazing on the same grassland (Yamamura et al., 2013). Thus, we suggest combining “grazing ban” with traditional nomadism to avoid excessive use of unmanaged grassland. The fence should be removed according to the vegetation restoration status. The study on the Tibetan plateau showed that degraded grassland can be restored after 4 to 8 years of fencing (Sun et al., 2020); thus, grazing-banned areas should be adjusted after such a period in order to avoid the negative edge effect.

## 5 Conclusion

The soil microbial and plant communities in the edge zone of grazing-banned grassland have been similar to those in adjacent unmanaged overgrazed grassland. The abundance of microorganisms was related to nutrient cycling reduced in both unmanaged and adjacent grazing-banned grasslands. The plant diversity, dominant plant species, soil organic carbon, and water content can explain the soil microbial community variation in both unmanaged and adjacent grazing-banned grasslands. Changes in soil water and organic carbon contents in unmanaged grassland affect these soil properties in adjacent grazing-banned grassland and subsequently affect soil microbial functions and plant community. Degradation in unmanaged grassland interferes with the restoration of grazing-banned grassland via negative edge effects.

## Data availability statement

Microbial raw sequence data that support the findings of this study are openly available in NCBI at Sequence Read Archive (SRA), reference number PRJNA1047307 (bacteria) and PRJNA1047293 (fungi).

## Author contributions

MF: Investigation, Software, Writing – original draft. GL: Writing – review & editing, Investigation. SZ: Writing – review & editing, Conceptualization, Supervision. WL: Writing – review & editing, Conceptualization.
